# Supplementary material for: Two-dimensional heavy fermion in a monoatomic-layer Kondo lattice YbCu2
Source: Nat Commun. 2023 Dec 1;14:7850. doi: 10.1038/s41467-023-43662-9 (PMC10692116; doi:10.1038/s41467-023-43662-9)
Supplement: Supplementary file 1 — Supplementary Information [file 41467_2023_43662_MOESM1_ESM.pdf]

Supplementary Information for: Two-dimensional heavy fermion in a monoatomic-layer Kondo lattice $YbCu_2$

Takuto Nakamura,[1,2,*] Hiroki Sugihara,[2] Yitong Chen,[2] Ryu Yukawa,[3] Yoshiyuki Ohtsubo,[4] Kiyohisa Tanaka,[5] Miho Kitamura,[6] Hiroshi Kumigashira,[7] and Shin-ichi Kimura[1,2,5,†]

[1]*Graduate School of Frontier Biosciences, Osaka University, Suita 565-0871, Japan*

[2]*Department of Physics, Graduate School of Science, Osaka University, Toyonaka 560-0043, Japan*

[3]*Graduate School of Engineering, Osaka University, Suita 565-0871, Japan*

[4]*National Institutes for Quantum Science and Technology, Sendai 980-8579, Japan*

[5]*Institute for Molecular Science, Okazaki 444-8585, Japan*

[6]*Photon Factory, Institute of Materials Structure Science, High Energy Accelerator Research Organization (KEK), 1-1 Oho, Tsukuba 305-0801, Japan*

[7]*Institute of Multidisciplinary Research for Advanced Materials (IMRAM), Tohoku University, Sendai, 980–8577, Japan*

* nakamura.takuto.fbs@osaka-u.ac.jp

† kimura.shin-ichi.fbs@osaka-u.ac.jp

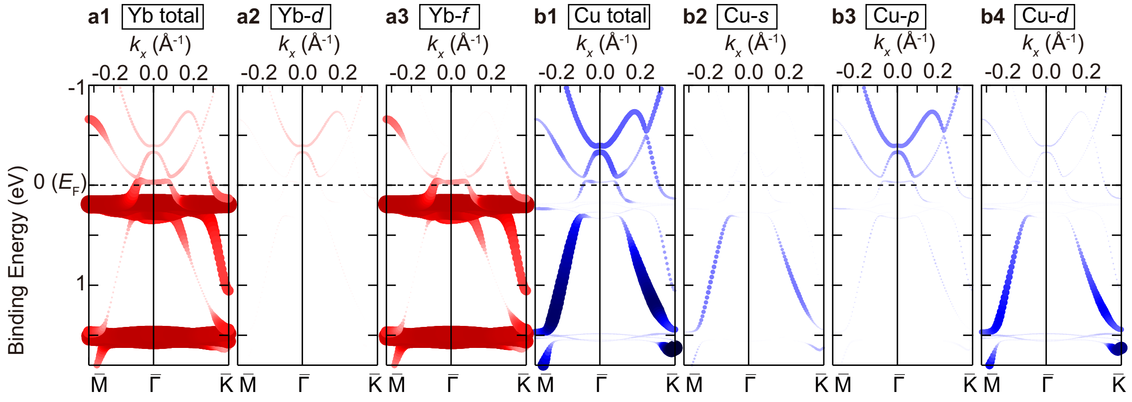

Supplementary Fig. 1. Orbitral contributions for the band structure of freestanding $YbCu_2$ by DFT calculation. (a1-3) Band structure of freestanding $YbCu_2$, where the radii of the circles represent the projected contribution of (a1) all Yb, (a2) f- and (a3) d-orbitals. (b1-4) same as (a) but for (b1) all Cu, (b2) s-, (b3) p- and (b4) d-orbitals.

SUPPLEMENTARY NOTE 1. ORBITAL CONTRIBUTIONS IN THE CALCULATED BAND STRUCTURE OF YBCU$_2$

In order to reveal the orbital character of the hybridized band, the band structure of the freestanding $YbCu_2$ is calculated as shown in Supplementary Fig. 1. The flat bands at the binding energy of 0.2 and 1.5 eV are derived from Yb $4f$ orbitals. The hole band with the $\bar{\Gamma}$ point at its apex is mainly derived from Cu with a small contribution from the Yb $5d$ state, which is consistent with the previous study [1–3].

The calculated band structures of the $YbCu_2$ on the Cu(111) substrate were shown in Supplementary Fig. 2. Overall shapes of the band structure are qualitatively consistent with the observed ARPES images as shown in Supplementary Fig. S2(c). The atomic orbitals in the innermost hole bands are mainly contributed from the Cu atoms in the $YbCu_2$ layer, indicating that these bands originate from the monolayer $YbCu_2$. Note that other hole bands with the $\bar{\Gamma}$ point at its apex are derived from Cu atoms in the substrate. These results indicate that the band structure of the monoatomic layered $YbCu_2$ still remains even including the Cu(111) substrate.

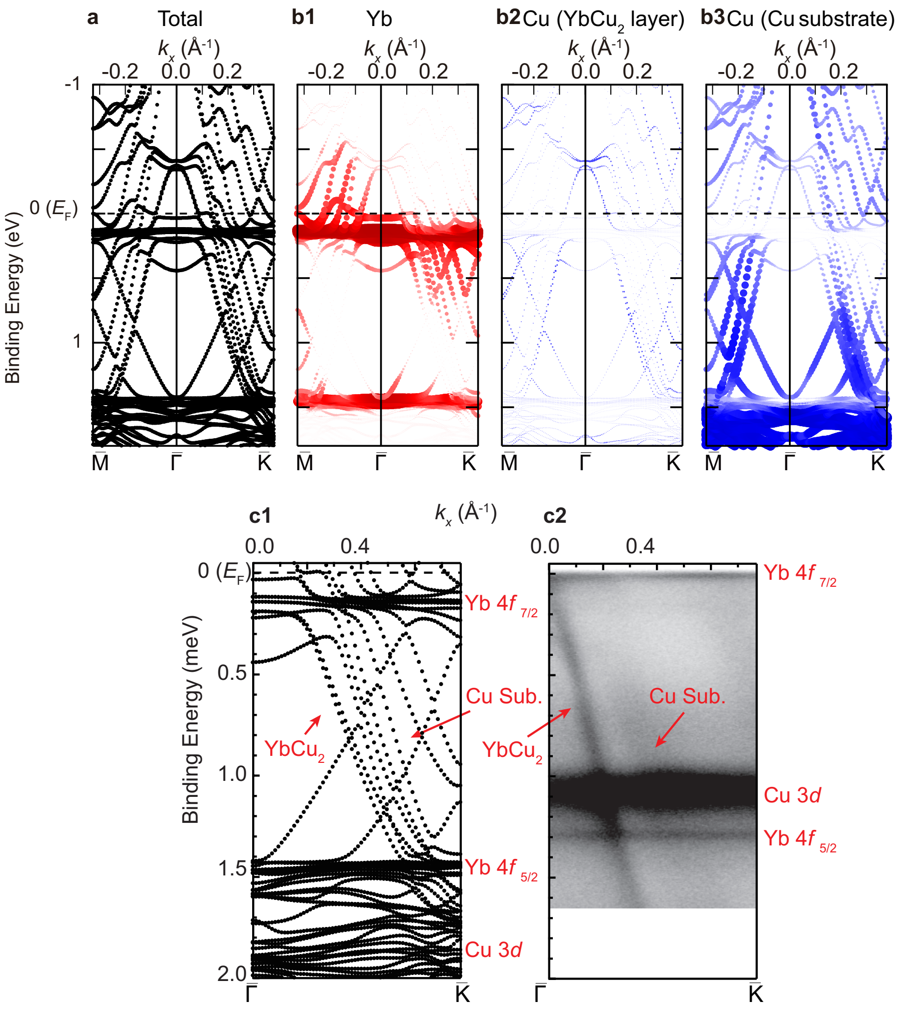

Supplementary Fig. 2. (a) Calculated band structure of the $YbCu_2$/Cu(111) slab. (b) same as (a) but the radii of the circles represent the projected contribution of (b1) Yb atom, (b2) Cu atom in the $YbCu_2$ layer, and (b3) Cu atom in the substrate. (c) The comparison of electronic structure between calculation and ARPES in the same energy and wavenumber scales. (c1) Calculated band structure of the $YbCu_2$/Cu(111) slab. (c2) ARPES intensity plots along $\bar{\Gamma}$–$\bar{\mathrm{K}}$ and with horizontally polarized 37-eV photons at 7 K. Yb 4f spin-orbit pair at 0.1 and 1.5 eV in the calculation is shifted to the low energy side in ARPES, and Cu 3d flat band at $E_B = 1.7$ eV in the calculation is also shifted to at 1.1 eV in ARPES originating from a self-energy effect.

SUPPLEMENTARY NOTE 2. PHOTON-ENERGY DEPENDENCE OF MDCS

Supplementary Fig. 3(a) shows the momentum distribution curves (MDCs) at $E_{\rm F}$ as a function of the excited photon energy. The peaks at the $k_x = \pm\, 0.08$ Å^{-1}, which correspond

to the Fermi wavenumber of the hybridized conduction band S1 in Fig. 3(c) in the main text, are constant with changing the excited photon energy, indicating that the hybridized band is two dimensional. Note that the peaks in MDCs excited at energies higher than 60 eV are not clearly visible due to the overlapping with other 3D hole bands as shown in Supplementary Fig. 2(b3). In order to confirm the contribution to the 2D HF state from the other hole band S3, which is the three-dimensional (3D) band described by the DFT calculation in Supplementary Fig. 1, the photon-energy dependence of MDCs at the normal emission at binding energies of 100 meV is shown in Supplementary Fig. 3(c). The peaks at $k_x = \pm$ 0.2 Å^{-1} in Fig. S3(b) depend on the excited photon energy suggesting the 3D character. This is consistent with the DFT calculations in Supplementary Fig. 2(b3). Note that the upper and lower branches of the c-f hybridization band (S1 and S2 bands in Supplementary Fig. 3(c)) have no photon-energy dependence suggesting the 2D character, which corresponds to Fig. 3(d) in the main text.

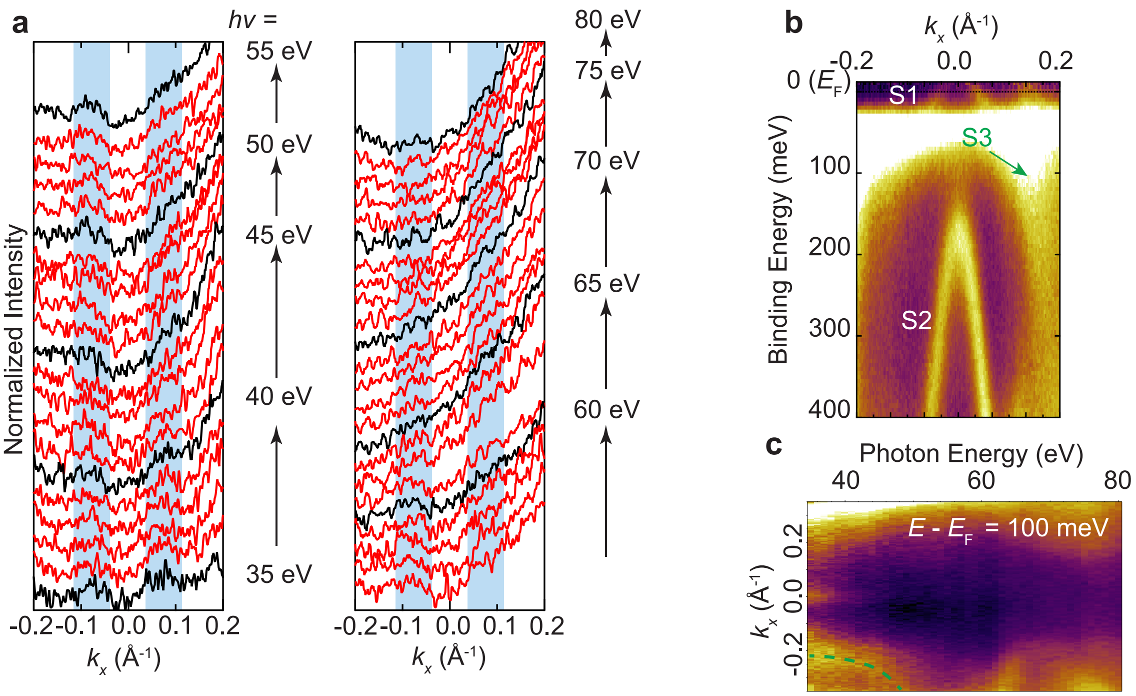

Supplementary Fig. 3. Momentum distribution curves (MDCs) at E_{F} with the energy windows of ± 10 meV at 15 K. The incident photon energies ranging from 35 to 80 eV. The intensities of all spectra are normalized by the average value of the peak at $k_x = \pm\ 0.1$ Å^{-1} to emphasize the peak energy position. The shaded area highlights the peak position of the MDCs. (b) Magnified ARPES image near the $\bar{\Gamma}$ point taken with circularly polarized 35-eV photons at 15 K. ARPES intensities are divided by the Fermi-Dirac distribution function convolved with the instrumental resolution. (c) Photon-energy dependence of MDCs at the normal emission at binding energies of 100 meV with the energy windows of ± 10 meV. Dashed lines indicate the guide of the MDC peak position by eye.

SUPPLEMENTARY NOTE 3. FITTING OF KONDO-RESONANCE PEAK IN PHOTOELECTRON SPECTRA

Supplementary Fig. 4 shows the temperature-dependent Kondo resonance peak with the fitting curves. All spectra are fitted with a Lorentzian function (purple line, an example for 130 K) after subtracting the Shirley-type background (black dotted line). For the fitting of spectra at the low temperature at $k_x = 0.0$ Å^{-1} in Supplementary Fig. 4 (c), an additional Voight component was added to reproduce the peak at the binding energy of 180 meV (a down arrow), which originated from another 2D band S2 in Fig. 3 in the main text. The

ARPES intensity plots used to obtain Supplementary Fig. 4 are shown in Supplementary Fig. 5.

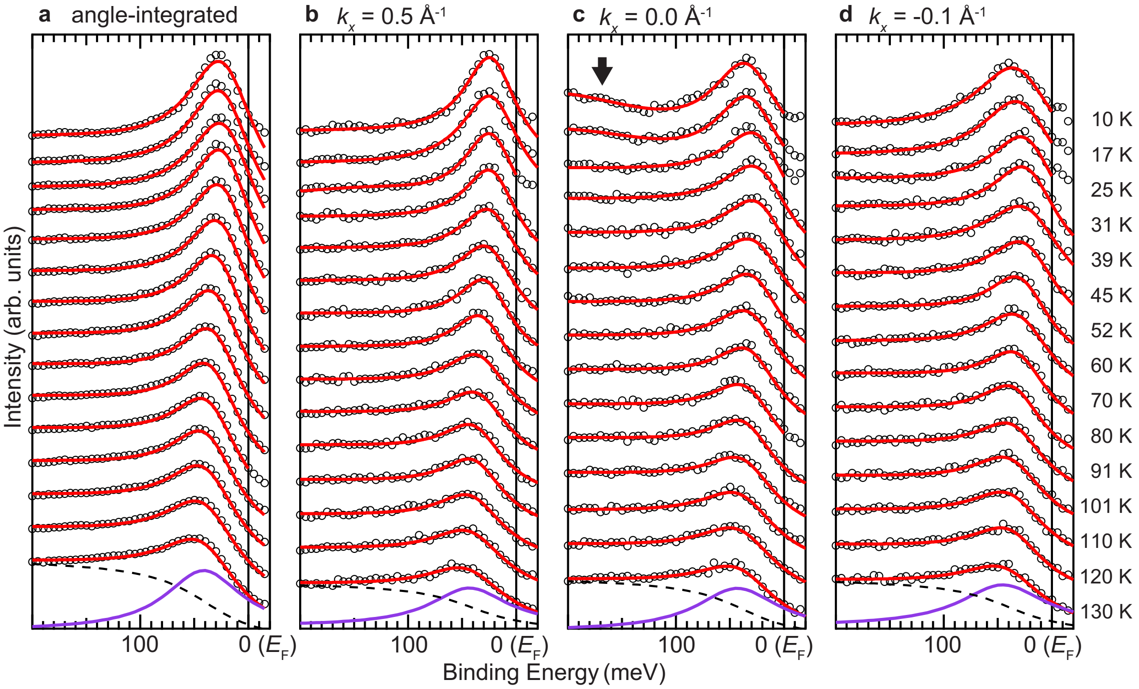

Supplementary Fig. 4. (a) Angle-integrated photoelectron spectra near E_F as a function of temperature taken with horizontally polarized 35-eV photons. (b-d) Angle-resolved photoelectron spectra near E_F as a function of temperature. The k_x positions at (b) 0.5, (c) 0.0, and (d) -0.1 Å^{-1} correspond to the local f state only, the $\bar{\Gamma}$ point, and the crossing point of the c-f hybridization, respectively. In all spectra, the intensity is normalized by the Fermi-Dirac distribution function convolved with the instrumental resolution. Purple lines indicate Lorentzian functions representing Kondo resonance peaks. The dashed lines show Shirley-type backgrounds.

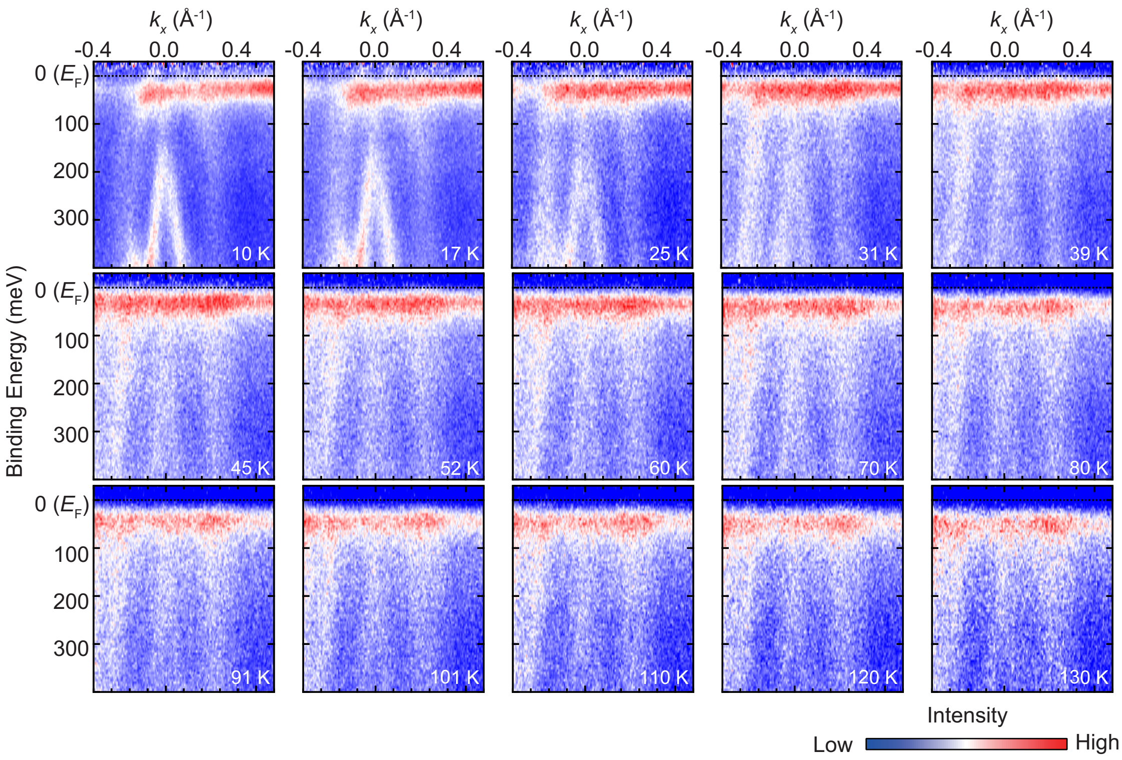

Supplementary Fig. 5. Temperature-dependent ARPES intensity plots measured with horizontally polarized 35-eV photons. ARPES intensities are divided by the Fermi-Dirac distribution function convolved with the instrumental resolution.

SUPPLEMENTARY NOTE 4. EXPERIMENTAL GEOMETRY OF ANGLE-RESOLVED PHOTOEMISSION SPECTROSCOPY (ARPES)

Supplementary Fig. 6 shows the geometry of the synchrotron-ARPES measurement in this study. Photon-incident angle is 45 to 50° respect to the normal of the photoelectron analyzer. Both the photoelectron detection plane and the photon-incident plane are on the xz plane. It should be noted that the positive and negative photoelectron emission angles are non-equivalent geometric configurations in this study.

SUPPLEMENTARY NOTE 5. EVALUATION OF c-f HYBRIDIZATION FEATURE BY PERIODIC ANDERSON MODEL

In order to confirm the validity of the bare bands by PAM analysis, an experimentally obtained ARPES image at room temperature is shown in Supplementary Fig. 7 overlayed

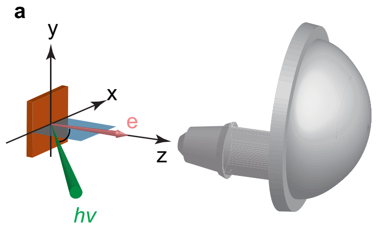

Supplementary Fig. 6. Schematic drawing of ARPES setup.

with assumed band dispersions with $V_k = 0$ meV. The shape of the assumed bare conduction band as well as the energy position of the $4f$ band is in good agreement with the ARPES image.

SUPPLEMENTARY NOTE 6. ORIGIN OF THE HOLE BAND AT $\bar{\Gamma}$ POINT IN YBCU$_2$/CU(111)

The electronic state of the nominal Cu has an electron-like *sp*- band against the Γ point. On the other hand, the atom adsorbed on the (111) plane of the noble metal substrates such as Au, Ag, and Cu typically form a $(\sqrt{3} \times \sqrt{3})$R30° periodicity and seem similar hole-like bands at the Γ point. The hole-like band is derived from hybridization between adsorbed atoms and Cu. The others originate from the replica bands of bulk *sp*-band and the Umklapp scattering of *sp*-band from $(\sqrt{3} \times \sqrt{3})$R30° periodicity. Our DFT calculation also suggests that the inner hole band in this study mainly consists of the topmost $YbCu_2$ layer, and it shows the mixture of orbitals not only Yb 5*d*- orbitals but also Cu *sp*- and *d*- orbitals, which is a similar situation for other noble-metal $(\sqrt{3} \times \sqrt{3})$R30° systems.

---

[1] Ormaza, M. *et al.* High Temperature Ferromagnetism in a $GdAg_2$ Monolayer. *Nano Lett.* **16**, 4230–4235 (2016).

[2] Fernandez, L. *et al.* Influence of 4f filling on electronic and magnetic properties of rare earth-Au surface compounds. *Nanoscale* **12**, 22258 (2020).

[3] Xu, C. *et al.* A two-dimensional $ErCu_2$ intermetallic compound on Cu(111) with moiré-pattern-modulated electronic structures. *Phys. Chem. Chem. Phys.* **22**, 1693–1700 (2020).

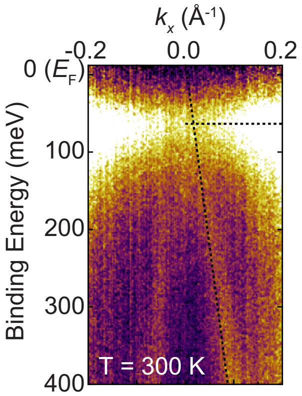

Supplementary Fig. 7. Magnified ARPES image near the $\bar{\Gamma}$ point taken with circularly polarized 35-eV photons at room temperature (300 K). ARPES intensities are divided by the Fermi-Dirac distribution function convolved with the instrumental resolution. The dashed lines indicate the assumed band dispersions ${E_k}^{\pm}$ with $V_k = 0$ meV in the PAM analysis.
